# Medical cannabis patterns of use and substitution for opioids & other pharmaceutical drugs, alcohol, tobacco, and illicit substances; results from a cross-sectional survey of authorized patients

**DOI:** 10.1186/s12954-019-0278-6

**Published:** 2019-01-28

**Authors:** Philippe Lucas, Eric P. Baron, Nick Jikomes

**Affiliations:** 1 Social Dimensions of Health, University of Victoria, 3800 Finnerty Rd., Victoria, BC V8P 5C2 Canada; 2 Tilray, 1100 Maughan Rd, Nanaimo, BC V9X1J2 Canada; 3 Canadian Institute for Substance Use Research, 2300 McKenzie Ave, Victoria, BC V8N 5M8 Canada; 4 Cleveland Clinic Neurological Institute, Department of Neurology, Center for Neurological Restoration - Headache and Chronic Pain Medicine , 10524 Euclid Avenue, C21, Cleveland, OH 44195 USA; 5 Principal Research Scientist; Division of Data Science, Leafly, 71 Columbia Street, Suite 200, Seattle, WA 98104 USA

**Keywords:** Cannabis, Opioids, Substitution, Addiction, Marijuana, Harm reduction

## Abstract

**Background:**

A 239-question cross-sectional survey was sent out via email in January 2017 to gather comprehensive information on cannabis use from Canadian medical cannabis patients registered with a federally authorized licensed cannabis producer, resulting in 2032 complete surveys.

**Methods:**

The survey gathered detailed demographic data and comprehensive information on patient patterns of medical cannabis use, including questions assessing the self-reported impact of cannabis on the use of prescription drugs, illicit substances, alcohol, and tobacco.

**Results:**

Participants were 62.6% male (*n* = 1271) and 91% Caucasian (*n* = 1839). The mean age was 40 years old, and pain and mental health conditions accounted for 83.7% of all respondents (*n* = 1700). Then, 74.6% of respondents reported daily cannabis use (*n* = 1515) and mean amount used per day was 1.5 g. The most commonly cited substitution was for prescription drugs (69.1%, *n* = 953), followed by alcohol (44.5%, *n* = 515), tobacco (31.1%, *n* = 406), and illicit substances (26.6%, *n* = 136). Opioid medications accounted for 35.3% of all prescription drug substitution (*n* = 610), followed by antidepressants (21.5%, *n* = 371). Of the 610 mentions of specific opioid medications, patients report total cessation of use of 59.3% (*n* = 362).

**Conclusions:**

This study offers a unique perspective by focusing on the use of a standardized, government-regulated source of medical cannabis by patients registered in Canada’s federal medical cannabis program. The findings provide a granular view of patient patterns of medical cannabis use, and the subsequent self-reported impacts on the use of opioids, alcohol, and other substances, adding to a growing body of academic research suggesting that increased regulated access to medical and recreational cannabis can result in a reduction in the use of and subsequent harms associated with opioids, alcohol, tobacco, and other substances.

## Background

Cannabis is the most widely used illicit substance in the world [1, 2]. Its use has been associated with both personal and social benefits as well as harms [2, 3]. While research supports the therapeutic use of cannabis in the treatment of chronic pain and other conditions [4–8], a comprehensive examination of the public health and safety impacts of cannabis needs to consider the growing body of evidence suggesting its use affects the use of potentially more problematic legal substances like alcohol and tobacco, and illicit substances like cocaine, heroin, and other opioids.

Bachhuber et al. (2014) report that US states with medical cannabis laws had a 24.8% lower mean annual opioid overdose mortality rate compared to states without medical cannabis laws (95% CI − 37.5% to − 9.5%, *p* = .003), and this trend strengthened over time. An examination of opioid positivity in fatally injured drivers in the USA found that state-based medical cannabis programs were associated with lower rates among 21 to 40 year olds, with the authors concluding that regulated access to medical cannabis may reduce opioid use and associated overdoses [9]. More recently, a cross-sectional study of state implementation of medical and adult-use cannabis laws from 2011 to 2016 found that medical cannabis laws were associated with a 5.88% lower rate of opioid prescribing [10].

Additional population-level research has explored how the recent introduction of regimes for legal access to cannabis (e.g., medical and/or recreational) in some US states has preceded reductions in homicides and violent crime [11], suicides [12], and automobile-related fatalities [13, 14], with all theorizing that these public health and safety benefits are related to subsequent declines in alcohol use.

However, cannabis use is not without potential harms. Chronic use has been associated with potential neurocognitive impairments particularly in adolescent use, and the psychoactive effects of use and associated impairment can lead to increased personal health and public safety risks associated with driving [15, 16]. While evidence suggests that smoking as a route of administration may increase the risk of bronchial issues [17, 18], the evidence in regards to associations between cannabis smoking and cancer is inconclusive. While some research has found a moderate increased risk of lung cancer in heavy users [19, 20], other studies have concluded that no causal association exists [17, 18, 21, 22]. It is important to note that the literature describing adverse events of cannabis including cardiovascular or cerebrovascular events is based on cannabis use from the black market without standardized quality control. Thus, these reports are based on cannabis of unknown quality, toxins, heavy metals, fungal/bacterial elements, strains, cannabinoid and terpene profiles, illicit drug use (laced cannabis or simultaneous use), concurrent prescription drug use, underlying patient medical history such as vascular risk factors, and many other cofounders which can influence adverse events.

There may also be some vulnerable populations in which risks are increased, such as women who may be pregnant, although the limited literature examining impacts of cannabis use on pregnancy is clouded by contradictory findings [23, 24]. Youth may be at greater risk since cannabis may have more significant cognitive and psychological impacts on developing brains [25, 26], and those with a pre-disposition for psychosis or schizophrenia may be particularly vulnerable since cannabis may perpetuate and potentially aggravate these conditions [27–29].

Social harms associated with cannabis include the impacts of impairment on driving [30, 31], and financial impacts on health care and police, court and incarceration costs resulting from prohibition [16, 32–34]. Some of the health-related harms and associated costs may be increased by the current international prohibition on cannabis, which leaves control over cannabis production and distribution to the black market. This criminal justice approach to cannabis regulation results in the criminalization of end-users, and consumers purchasing cannabis products of unknown potency, with no quality controls or age restrictions on access [2, 35, 36], making it challenging to study the actual impacts of cannabis use on individuals without significant confounders related to both prohibition and the black-market control over production and distribution.

Recently, these negative policy-related outcomes have led many jurisdictions to try alternative regulatory approaches, including the legalization of medical cannabis use in Canada and much of Latin American and the European Union, and the regulation of recreational adult use in a number of US states, Canada, and Uruguay. This study provides a unique perspective by focusing on the use of a standardized, government-regulated source of medical cannabis by patients registered in Canada’s federal medical cannabis program. The findings provide a more granular view of patient patterns of medical cannabis use, and the subsequent self-reported impacts on the use of opioids, alcohol and other substances.

### Medical cannabis in Canada

In response to legal recognition of the constitutional rights of Canadians to access cannabis for medical purposes, in 2001, Canada became one of the first nations in the world to establish a national medical cannabis program [37, 38]. The Marihuana Medical Access Regulations (MMAR) allowed patients with chronic or incurable medical conditions like cancer, MS, HIV/AIDS, and severe chronic pain to apply to Health Canada for permission to possess a limited amount of medical cannabis, and in 2003 Health Canada began to provide patients with access to a legal source of flower cannabis consisting of a single cultivar produced under a federal contract and shipped to patients via mail [37].

The program has undergone numerous changes since its initiation due to shifts in policy and in response to successful legal challenges by patients who found the program inadequate in addressing their medical needs [39, 40]. This culminated in the establishment of the Marihuana for Medical Purposes Regulations (MMPR) in 2014, and ultimately in the Access to Cannabis for Medical Purposes Regulations (ACMPR) in August 2016 [37]. One of the primary changes of the MMPR/ACMPR was the authorization of multiple licensed producers (LPs) of cannabis. Under the ACMPR, patients get cannabis recommendations from physicians or nurse practitioners without the past restrictions on medical conditions found in the MMAR, and can then grow a limited amount of their own cannabis and/or access it from LPs, who ship it directly to patients via mail [38, 41]. As of September 2018, there were over 130 federally authorized licensed producers providing hundreds of strains of cannabis as well as cannabis extracts like oils or capsules to approximately 342,103 patients [42]. Under the ACMPR, health care providers (HCPs) make a written recommendation of the amount of cannabis that patients are authorized to use and ultimately possess in “grams per day”; it is the responsibility of each LP to determine the quantity of cannabis oil that is equivalent to 1 g of dried cannabis [42]. While some HCPs provide restrictions in regards to the potency or type of cannabis patients may order from LPs and subsequently use, no condition-specific clinical guidelines have been developed yet, and patients are typically free to order and use whatever type of cannabis (i.e., specific cultivars, potencies, or types of cannabis products) they feel may work best in addressing their medical conditions or symptoms [37, 41, 43].

As part of the ACMPR, Health Canada keeps detailed records of the amount of cannabis produced and distributed to patients; however, there is very little data on what conditions or symptoms affect these patients, how they use cannabis products (i.e., type of product/mode of use, frequency and dosages), and the impact of that use on the use of prescription medications, alcohol, tobacco, and other substances.

## Methods

In the interest of learning more about why/how patients use medical cannabis and whether this use affects the use of other substances, a 239-question patient feedback questionnaire was designed to gather comprehensive information on patient demographics, patterns of cannabis use, access to cannabis, and cannabis substitution effect from 2000 Canadian medical cannabis patients registered with Tilray, a federally authorized medical cannabis production, distribution, and research company located in Nanaimo, BC. This presented a unique cohort of cannabis users to assess, since they accessed a quality-tested, standardized cannabis supply under the auspice of a legal, tightly regulated medical cannabis regime.

The primary objectives of the Tilray Patient Survey 2017 were to:

1. Describe the characteristics of the sample, including patient demographics, use of cannabis, prescription drugs, alcohol, tobacco, and illicit substances.
2. Assess the extent to which cannabis is used as a substitute for prescription drugs, alcohol, tobacco, and illicit substances, and differences in the extent of substitution among the most frequently used drugs.
3. Assess the extent to which substitution is mediated by age, gender, primary medical condition/symptoms, cannabis strains, and method of use.

This survey was ethics reviewed and approved by Advarra (formerly Institutional Review Board Services), and the University of Victoria subsequently approved a post-survey sub-analysis of the substitution data. Data gathering was done on REDCap, a HIPAA, and PIPEDA compliant electronic data capture system. HIPAA (Health Insurance Portability and Accountability Act of 1996) is US legislation that provides data privacy and security provisions for safeguarding medical information, and PIPEDA is the Canadian federal privacy law governing private-sector organizations.

Participants were adult federally authorized medical cannabis patients registered with Tilray that provided the company with an email address, were capable of reading and understanding French or English, and were able to legally consent to participate in the study.

A password-protected link to an online survey available in French and English was sent out to 16,675 Tilray patients. Clicking on the link in the email invitation took patients to the IRB-approved informed consent form (ICF), and consent was gathered electronically. All questions were set to “must provide value”, so all questionnaires were fully answered with the exclusion of questions not relevant to individual respondents (i.e., if patients never used tobacco, then questions re. tobacco substitution were programmed not to appear via REDCap’s “skip logic” function). To compensate patients for taking the time to respond and share their experiences, Tilray offered a $10 credit on future cannabis purchases to all patients who completed the survey. The study budget of $20,000 allowed for the gathering of up to 2000 responses, and the survey deliberately closed after gathering 2032 complete responses (32 participants were in the process of completing the survey when this cut off was met so they were permitted to finish, resulting in 2032 complete surveys).

### Measures

The survey was composed of multiple choice, ranking, and open-ended questions to gather demographic data, health-related information, and details on medical cannabis use and its impact on the use of other substances. A question regarding primary condition provided a selection of 21 common medical conditions for which patients chose to use cannabis, and participants could only click one box (or click *other* and provide details in a text box), but a follow-up question regarding primary symptoms allowed participants to click multiple boxes from a selection of 13 common symptoms (or to click *other* and provide details in a text box). This allowed for a broader analysis of why patients specifically use medical cannabis in the treatment of different conditions; for example a participant citing *MS* as a primary condition might report use of cannabis to address *insomnia*, *pain*, and *spasticity* under primary symptoms.

A comprehensive assessment of cannabis use included questions on frequency of use per day and per week; yesterday use; amount used per session of use, as well as per day and per week; methods of ingestion (*Vaporizer*—*cannabis flowers*/*bud*; *Oral* (*edibles such as oil drops*/*extracts*, *baked goods*, *butter*, *tincture*), *Joint*; *Pipe*; *Waterpipe*/*bong*; *Vaporizer*/*nail*/*vape pen*—*cannabis extracts* (*shatter*, *budder*, *oil*, etc.); *Topical* (*on the skin*; e.g., *lotions*/*salves*, etc.); *Juicing* (4, 0.2%); *Other* (*please specify*)); preferred cannabis flower variety as classified by Tilray (either *indica*, *sativa*, *hybrid*, *1*:*1 CBD*/*THC*, *3*:*1 CBD*/*THC*); and favorite cultivar/strain with a drop-down menu of all 37 cultivars or blended milled flower available from Tilray to that point, and an “other” option with an additional textual response. Those who acknowledged use of Tilray extract products (oil-based *drops* or *capsules*) were asked about their preferred type of extract as labeled by the producer (*indica*, *sativa*, *hybrid*, *1*:*1 CBD*/*THC*, *3*:*1 CBD*/*THC*), frequency of use per day, and whether or not they also used flower, and if so, what percentage of their use was flower vs. extract in a quintile range from 10% flower/90% extract to 10% extract/90% flower. Participants were also asked to rank their current level of cannabis knowledge from 0 to 100, with 1 being *no knowledge and experience at all*, and 100 being *very knowledgeable and experienced*.

In order to assess substitution, participants were asked if they “ever regularly used or currently use” any of the following four classes of drugs: *prescription drugs*, *alcohol*, *tobacco*, and *illicit substances*. Those who responded positively were then asked if they had ever used cannabis as a substitute for these substances. Those that responded positively to substitution for either prescription drugs or illicit drugs were then asked to name up to three specific drugs they substituted for, and then asked at what percent they substituted cannabis for each specific drug by choosing from the following options in a multiple choice, single-answer: 100% (Stopped using this drug completely); 75% (Reduced my use of this drug by 3/4); 50% (Reduced my use of this drug by half); 25% (Reduced my use of this drug by ¼).

For both alcohol and tobacco, participants who responded positively to substitution were then asked at what quartile percent they substituted via the same multiple choice used to assess the self-reported rate of substitution for prescription and illicit substances highlighted above.

### Analysis

Primary analysis for this study was done on SPSS. Objective 1 was assessed using descriptive statistics (frequencies) to describe the characteristics of the sample. The distribution of the qualitative responses for *other* in both the primary condition and primary symptoms questions were examined and recoded if they match with the listed conditions; otherwise, they were left as *other.* Amount of flower cannabis was determined by examining self-reported cannabis use per week in grams, and extract use by frequency of daily and weekly use (“times per day” × “days per week” use).

Objective 2 was assessed using descriptive statistics to quantify the degree of substitution based on quartiles from 25 to 100%. Several independent frequencies were conducted for the percent of substitution from 0 (recoded to 0, if no other percent is given) to 100, restricted only to those who reported any regular lifetime use for each substance. Analyses recoded the prescription drugs into logical sub-categories (i.e., opioids, benzos, antidepressants, muscle relaxants, etc.), as well as the illicit substances (i.e., cocaine/crack cocaine, opioids, stimulants, psychedelics, etc.).

Objective 3 was assessed using bivariate logistic regression analyses for substitution effect of each major drug (i.e., opioids, alcohol, tobacco, and illicit substances), which were recorded as dichotomous variables (DVs). Medical condition/symptoms (i.e., pain, mental health) were reduced into a smaller number of logical groupings—*arthritis*/*musculoskeletal* and *headache* were grouped with *chronic pain* to form a broader chronic pain category, and *PTSD* was grouped with *mental health* to create a broader mental health primary condition category, and *stress*, *anxiety*, *depression* were grouped to create a broader primary symptoms category—and treated as categorical IVs. Similarly, cannabis flower varieties and extracts were grouped to best reflect THC/CBD ratios ranging from “high THC/low CBD” to “High CBD/low THC”.

## Results

The mean age of the sample was 40 years old, 1271 of which identified as *male* (62.6%), 758 *female* (37.3%), and 3 as *Other* (non-binary, transmasculine) (0.15%). In terms of marital status and racial backgrounds, 54.5% were *married or in domestic partnership* (*n* = 1107), and 91% (*n* = 1839) identified as *Caucasian*; 6% [122] *Metis*/*First Nations*, 2% [37] *South Asian*, and 2% [35] *Asian*. This was a highly educated population compared to the Canadian General Population (CGP); 93% (*n* = 1893) of participants had a *high school degree* (compared to 87% CGP) and 22% (*n* = 444) had a *university degree or higher* (compared to 17% of CGP) [44]. In regards to employment status, 63% of participants worked *full time* or *part time* (*n* = 1276), and 19% were *disabled/not able* to work (*n* = 384). In terms of income, participants were nearly identical to the CGP: 6.6% made less than $10,000 (*n* = 135; CGP = 5%), 25.6% made $10,000–$39,999 (*n* = 521; CGP: 27%), 42% made $40,000–$99,999 (*n* = 852; CGP: 42%), and 26% made $ > 100,000 (*n* = 524; CGP: 26%) [44].

### Primary condition and symptoms

In terms of primary condition and primary symptoms, pain and mental health conditions were dominant, as seen in Table 1 and Table 2, respectively. For primary condition, *chronic pain* was reported by 29.4% (*n* = 598), and *mental health condition* was reported by 27% (*n* = 548). This was followed by *insomnia* (9.7%, *n* = 198), *arthritis*/*musculoskeletal* (9.3%, *n* = 188), *PTSD* (4.6%, *n* = 93), and *headache* (3.7%, *n* = 75). In all, pain and mental health conditions—*PTSD*, *mental health*, *insomnia*, *chronic pain*, *arthritis*, *headache*—accounted for 83.7% of all respondents (*n* = 1700).

**Table 1:** Primary conditions treated with medicinal cannabis

Primary conditions	Total: *n* (%)	Male: *n* (%)	Female: *n* (%)	Unspecified: *n* (%)
Total study population	2032	1271 (62.6%)	758 (37.3%)	3 (0.15%)
Chronic pain	598 (29.4%)	371 (62%)	227 (38%)	
Mental health condition	548 (27%)	319 (58.2%)	228 (41.6%)	1 (0.2%)
Insomnia	198 (9.7%)	145 (73.2%)	53 (26.8%)	
Arthritis/musculoskeletal	188 (9.3%)	112 (59.6%)	76 (40.4%)	
PTSD	93 (4.6%)	59 (63.4%)	33 (35.5%)	1 (1.1%)
Headache	75 (3.7%)	44 (58.7%)	31 (41.3)	
Gastrointestinal disorder	62 (3.1%)	34 (54.8%)	28 (45.2%)	
Multiple sclerosis	45 (2.2%)	26 (57.8%)	19 (42.2%)	
Other	38 (1.9%)	23 (60.5%)	15 (39.5%)	
Cancer/leukemia	35 (1.7%)	24 (68.6%)	11 (31.4%)	
Crohn’s disease	35 (1.7%)	27 (77.1%)	8 (22.9%)	
Brain injury	24 (1.3%)	16 (66.7%)	8 (33.3%)	
Epilepsy/seizure disorder	21 (1.0%)	18 (85.7%)	3 (14.3%)	
Eating disorder	20 (1.0%)	10 (50%)	10 (50%)	
Diabetes	16 (0.79%)	13 (81.3%)	3 (18.7%)	
Movement disorder	10 (0.49%)	8 (80%)	1 (10%)	1 (10%)
AIDS/HIV	8 (0.39%)	7 (87.5%)	1 (12.5%)	
Hepatitis	6 (0.30%)	6 (100%)	0 (0%)	
Glaucoma	5 (0.25%)	5 (100%)	0 (0%)	
Osteoporosis	4 (0.20%)	3 (75%)	1 (25%)	
Skin condition	3 (0.15%)	1 (33.3%)	2 (66.7%)

**Table 2:** Primary symptoms treated with medicinal cannabis

Primary symptoms	*n* (%)
Total study population	2032
Chronic pain	1184 (58.3%)
Anxiety	991 (48.8%)
Insomnia	939 (46.2%)
Stress	929 (45.8%)
Depression	767 (37.8)
Headache	505 (24.9%)
Appetite loss	371 (18.3%)
Nausea	343 (16.9%)
Spasms	241 (11.9%)
Gastrointestinal disorder	239 (11.8%)
Memory loss	83 (4.1%)
Other	49 (2.4%)
Seizures	48 (2.4%)
Intraocular eye pressure	23 (1.1%)

In regards to primary symptoms, *chronic pain* was reported by 58.3% (*n* = 1184), followed by *anxiety* (48.8%, *n* = 991), *insomnia* (46.2%, *n* = 939), *stress* (45.8%, *n* = 929), and *depression* (37.8%, *n* = 767).

### Cannabis use

In regards to cannabis use, 74.6% (*n* = 1515) reported daily use, with a median of twice a day use (25.1%, *n* = 511). Across all primary conditions, the majority of patients reported using cannabis on a daily basis (7 days/week), as seen in Table 3. Mean amount used per day was 1.5 g, with 78.5% (*n* = 1595) of participants reporting they use less than 3 g per day, and 9.9% (*n* = 201) reporting they use 4 g or more per day.

**Table 3:** Frequency of daily cannabis use across all primary conditions

Primary condition	Total (*n*)	Daily cannabis use (*n*)	Daily cannabis use percentage (%)
Osteoporosis	4	4	100.0%
Wasting syndrome	1	1	100.0%
Epilepsy/seizure Disorder	21	19	90.5%
Crohn’s disease	35	30	85.7%
PTSD	93	79	85.0%
GI disorder	50	40	80.0%
Movement disorders	5	4	80.0%
Brain injury	24	19	79.2%
Chronic pain	598	453	75.8%
MS	45	34	75.6%
AIDS/HIV	8	6	75.0%
Diabetes	16	12	75.0%
Eating disorder	20	15	75.0%
Mental health condition	529	392	74.1%
Cancer/leukemia	34	25	73.5%
Arthritis	188	136	72.3%
Insomnia	191	135	70.7%
Glaucoma	5	3	70.0%
Hepatitis	6	4	66.7%
Other	82	54	65.9%
Headache	75	49	65.3%
Skin condition	2	1	50.0%

In regards to primary method of use, *vaporizer* (*cannabis flowers*/*bud* or *nail*/*pen*) was cited by 31.1% (*n* = 632), followed by *joint* (30.4%; *n* = 617), *oral*/*edible* (16.3%, *n* = 332), *pipe* (11.3%, *n* = 229), *waterpipe*/*bong* (10.4%, *n* = 212), *topical* (0.3%, *n* = 6), and *juicing* (0.2%, *n* = 4). This means that while inhalation via vaporization and/or smoking was by far the most popular methods of use, accounting for 83.2% (*n* = 1690), non-smoked methods of use (*oral ingestion*, *topical*, and *vaporization*) accounted for nearly half (47.6%, *n* = 968) of the primary methods of use.

In regards to flower cannabis (“bud”), 32% preferred *hybrids* (*n* = 651), 28% preferred *indicas* (*n* = 569), 24.7% preferred *sativas* (*n* = 502), and 14.8% preferred high cannabidiol (CBD) strains (1:1 CBD/THC or greater CBD content) (*n* = 300), with 0.49% (*n* = 10) citing *no preference*. This suggests that when smoking or vaporizing cannabis—which is what patients do with cannabis flower—a significant majority (84.7%; *n* = 1722) prefer strains high in THC.

However, this was not the case with orally ingested oils/extracts. Overall, 38.5% of respondents used orally ingested cannabis oils/extracts (*n* = 782), with 49.2% citing they mostly used high CBD oils/extracts (*n* = 381). Oils/extracts were used with a lower frequency than flower, with only 29.5% reporting daily use (*n* = 226), and the median frequency of use per day being once per day (65.6%; *n* = 497). Additionally, 66.5% of those who reported using oil/extracts also used flower on the same day (*n* = 514), with a breakdown of 10% extract/90% flower for 45.1% (*n* = 230), 25% extract/75% flower for 23.3% (*n* = 119), 50% extract/50% flower for 16.5% (*n* = 84), 75% extract/25% flower for 9.6% (*n* = 49), and 90% extract/10% flower for 5.5% (*n* = 28). Therefore, for most participants, oils/extracts appear to be adjunct treatments to the smoking or vaporization of cannabis flower.

When participants were asked to rank their current level of cannabis knowledge from 0 to 100 (with 1 being *no knowledge and experience at all*, and 100 being *very knowledgeable and experienced*), the mean was 73.65 (SD21.81). Interestingly, neither age nor income were significantly associated with different levels of knowledge, but there was a significant linear correlation (*r* = 0.29) with grams consumed per week and self-reported knowledge level, and higher knowledge levels were more likely to use *vape pens* and *water bongs*, while lower levels of knowledge were more likely to use *oral*/*extracts* (*p* > .05), suggesting that naïve or less experienced cannabis users may have had a greater level of comfort with non-inhaled, orally-ingested modes of ingestion.

### Substitution for other substances

The most commonly cited substitution was for *prescription drugs* (69.1%; *n* = 953), followed by *alcohol* (44.5%; *n* = 515), *tobacco* (31.1%; *n* = 406), and *illicit substances* (26.6%; *n* = 136), as seen in Table 4. Overall, women were more likely to report substitution than men (*p* < .01; 95% CI).

**Table 4:** Drugs substituted with cannabis

Substituted drugs	Patients who reported drugs used (*n*)	Cannabis used as a substitute (*n*, %)
1. Prescription drugs	1379	953; 69.1%
	M: 871; 63.2%	M: 613; 64.3%
	F: 505; 36.6%	F: 338; 35.5%
	U: 3; 0.2%	U: 2; 0.2%
2. Alcohol	1158	515; 44.5%
	M: 743; 64.2%	M: 348; 67.6%
	F: 413; 35.7%	F: 166; 32.2%
	U: 2; 0.2%	U: 1; 0.2%
3. Tobacco	1307	406; 31.1%
	M: 832; 63.7%	M: 268; 66%
	F: 473; 36.2%	F: 137; 33.7%
	U: 2; 0.2%	U: 1; 0.3%
4. Illicit substances	511	136; 26.6%
	M: 345; 67.5%	M: 93; 68.4%
	F: 165; 32.3%	F: 43; 31.6%
	U: 1; 0.2%	U: 0; 0%

*M* male, *F* female, *U* unspecified

Participants named a total of 1730 specific prescription drugs they substituted cannabis for, 35.3% (*n* = 610) of which were opioids, 21.5% (*n* = 371) anti-depressants, 10.9% (*n* = 189) non-opioid pain medications, 8.6% (*n* = 149) anti-seizure medications, 8.1% (*n* = 140) muscle relaxants/sleep aids, 4.3% (*n* = 75) benzodiazepines, 3.4% (*n* = 59) stimulants, 1.4% (*n* = 24) anti-emetics, and 1% (*n* = 18) anti-psychotics, as seen in Table 5. Of the 610 mentions of opioid medications, participants self-reported they stopped using 59.3% completely (100% substitution) (*n* = 362), and a further 18.4% reduced their use by 75% (*n* = 112). Participants who substituted for opioids used more cannabis per day (1.71 g vs. 1.46 g by those who did not cite substitution for opioids) and were also more likely to report extract/oral use as their primary method of use (21%; *n* = 458 vs. 15%; *n* = 1574; *p* = 0.01) and to use extracts on a daily basis (40%; *n* = 187 vs. 26%; *n* = 580; *p* = 0.004). In ranking reasons for substituting cannabis for prescription drugs, the reason ranked as the “most important” by the highest number of participants was the belief that *cannabis is a safer alternative than prescription drugs* (ranked #1 by 51.2%; *n* = 419), followed by *fewer adverse side*-*effects* (39.7%; *n* = 207), *better symptom management* (19.5%; *n* = 124), *fewer withdrawal symptoms* (11.4%; *n* = 61), *the ability to obtain cannabis* vs. *prescription drugs* (3%; *n* = 18), and *social acceptance of cannabis is greater than prescription drugs* (2.4%; *n* = 17).

**Table 5:** Breakdown of drugs substituted with cannabis

Prescription drugs (*n*, %)^a^	
1. Opiates/opioids	610; 35.3%
2. Anti-depressant/anti-anxiety	371; 21.5%
3. Non-opioid pain medications	189; 10.9%
4. Anti-seizure medications	149; 8.6%
5. Muscle relaxant/sleep aids	140; 8.1%
6. Benzodiazepines	75; 4.3%
7. Stimulants	59; 3.4%
8. Antiemetics	24; 1.4%
9. Antipsychotics	18; 1%
Illicit drugs (*n*, %)^b^	
1. Cocaine/Crack	89; 17.4%
2. Psychedelics	60; 11.7%
3. Non-prescription opioids	29; 5.7%
4. Stimulants	14; 2.7%
5. Depressants	8; 1.6%

^a^Out of 1730 specific prescription drugs substituted by cannabis

^b^Out of 511 illicit drugs substituted by cannabis

Of the 515 respondents who substituted cannabis for alcohol, 30.9% suggested they stopped using it completely (100%) (*n* = 159), and 36.7% reported reducing by at least 75% (*n* = 189). Participants who reported mental health symptoms were more likely to substitute for alcohol (*n* = 834; *p* = .0005), whereas participants who reported pain symptoms were less likely to substitute for alcohol (*n* = 761; *p* = .002).

Of the 406 participants who substituted cannabis for tobacco, 50.7% say they stopped using it completely (100% substitution) (*n* = 206), and 13.8% reported reducing their use by 75% (*n* = 56). Those who substituted for tobacco were far more likely to smoke cannabis as their primary method of use (66% vs. 48% of those who did not substitute for tobacco; *p* < .0001). A more detailed analysis of the tobacco substitution data from this study is the subject of a separate publication.

In regards to illicit substances, cocaine/crack was the most frequently substituted illicit drug (17.4%; *n* = 89), followed by psychedelics (11.7%; *n* = 60), non-prescription opioids (5.7%; *n* = 29), stimulants (2.7%; *n* = 14), and depressants (1.6%; *n* = 8), as seen in Table 5. Of those who cited substituting for illicit substances, 72.2% suggested they substituted at 100% for that specific drug mentioned (*n* = 332).

## Discussion

This study represents the largest polling of Canadian medical cannabis patients to date, and the first broad survey of cannabis patients that we are aware of that has gathered such a detailed inventory of cannabis substitution for other drugs, including the specific prescription and illicit substances being substituted for, and the self-reported rate of that substitution as identified in quartile percentages, significantly improving the overall understanding of medical cannabis use and its impact on the use of opioids, alcohol, and other substances.

The finding that cannabis was being used for the treatment of chronic pain is in keeping with past research [45, 46], but the growing use to treat mental health conditions like anxiety, stress, and depression suggests an emerging trend in medical cannabis use [38, 47]. Despite ongoing concerns in regards to the impact of cannabis use by those with a predisposition for psychosis and schizophrenia [47, 48], the use to address more minor but widespread mental health conditions represents a potential broadening of therapeutic applications and a good target for further research [47, 49, 50].

The high frequency of daily use (74.6%) may suggest an area of concern in terms of potential long-term impacts on physical and mental health, but it is likely a reflection of the high rate of use for pain and mental health conditions, both of which are chronic conditions with daily traditional pharmacological treatment options. While daily use may pose a risk of dependence in some patients, research suggests a low risk overall. In a 2015 report titled *State of the Evidence*; *Cannabis Use and Regulation*, the International Centre for Science in Drug Policy assessed the state of evidence regarding the potential harms of cannabis use. They found the lifetime use of cannabis carried a low risk of dependence (9%), while the lifetime risk of dependence to alcohol has been assessed at 22.7%, and the lifetime risk of heroin dependence is estimated to be between 23.1 and 35.5% [51].

Additionally, while 52.4% of respondents cited smoking as a primary route of administration, which could result in bronchial harms [18, 22], this data also suggests an ongoing trend away from the smoked ingestion of high THC products and toward vaporization and the oral ingestion (oils or capsules) of high CBD extracts (oil or capsules) [37, 38]. This is likely due to an increase in non-smoked product options available to patients via the ACMPR, a growing awareness of the potential therapeutic benefits of CBD (particularly in regards to the treatment of mental health conditions), and a more health-conscious approach to medical cannabis use overall. However, with more and more patients around the world using cannabis for medical purposes, prospective studies will be necessary to assess the long-term health implications of chronic daily medical use, inform therapeutic considerations by patients and health care providers, and develop evidence-based treatment guidelines.

This study’s findings on the self-reported reduction of opioid use are particularly significant to public health and safety. The past decade has seen the rapid growth of an opioid epidemic in North America, and currently drug overdose is the leading cause of accidental death in Canada and the US, with many of these deaths resulting from both prescription and illicit opioids. In 2015, there were 52,404 drug overdose deaths in the USA, including 33,091 (63.1%) overdose deaths related to opioids [72]. In Canada, it is estimated that 4000 people died of an opioid overdose in 2017, 1450 of which were in British Columbia alone [52], and opioid overdose now results in an average of 16 hospitalizations per day, a 53% increase in opioid-related hospitalizations over the last 10 years [53]. Cannabis may not only reduce the prescription and use of opioids in medical and non-medical users [45, 54–56], it may also reduce the odds of transitioning to more problematic substances and patterns of use. Reddon et al. (2018) found that daily cannabis use was associated with slower rates of injection drug initiation among street youth in Vancouver (adjusted relative hazard 0.66, 95% confidence interval 0.45–0.98; *p* = 0.038) [57]. Furthermore, recent evidence suggests that cannabis use may reduce opioid withdrawal and improve outcomes of opioid-replacement therapies (methadone/Suboxone) for those seeking treatment for opioid use disorder [57–60], thereby reducing the public health impacts of the current opioid overdose crisis.

Concurrent with the policy trend toward the regulation and legalization of both medical and adult recreational use in many US states, Canada, and around the world is an urgent need to develop mechanisms to systematically track both the positive and negative impacts of cannabis use on individuals and society. However, this study suggests that regulating access to a standardized, quality-controlled source of cannabis for authorized patients may provide an opportunity to both reduce the harms associated with cannabis use, and to maximize the potential positive impacts on public health and safety stemming from cannabis substitution effect. Future studies involving standardized, quality-controlled cannabis will also provide more accurate assessments of not only benefits of cannabis, but potential side effects and adverse events as well.

### Strengths and limitations

This study has a number of strengths and limitations. Budgetary restrictions resulted in deliberately cutting off the survey at 2032 participants, leaving open the possibility this could be an unrepresentative sample. Since this sample is drawn from a patient population registered with a medical cannabis company, respondents may be more likely to report positive effects related to the medical use of cannabis—including substitution effect—because of selection bias. All data in this study were self-reported retroactive data by patients and did not benefit from biological drug detection to confirm use or non-use of a substance, so is subject to potential recall bias. Since the questionnaire measured “ever” use of drugs and subsequent substitution, there is no way to determine whether self-reported substitution was a temporary or lasting outcome. However, the identification of specific prescription drugs substituted for by respondents partially mitigates this issue for this class of substances, since respondents specifically refer to substitution of an actual prescription drug, rather than a drug class like alcohol or tobacco. In light of these potential biases, the characterization of the therapeutic use of cannabis and/or cannabis substitution effect should be interpreted with caution pending replication by research that employs a more systematic recruitment approach, longitudinal monitoring, and biological drug testing.

These limitations are counterbalanced by several methodological strengths, including the large size of the sample, assurance that all participants were using a standardized, high-quality source of cannabis with the support of a health care practitioner, and adherence to established standards for reporting Internet-based surveys [61].

## References

[1] Russo EB (2007). History of cannabis and its preparations in saga, science, and sobriquet. Chem Biodivers.

[2] Fischer B, Russell C, Sabioni P, van den Brink W, Le Foll B, Hall W (2017). Lower-risk cannabis use guidelines: a comprehensive update of evidence and recommendations. Am J Public Health.

[3] Grinspoon L (2007). On the future of cannabis as medicine. Clin Trials.

[4] Ware MA, Wang T, Shapiro S, Collet J-P (2015). Cannabis for the management of pain: assessment of safety study (COMPASS). J Pain.

[5] Ware MA, Wang T, Shapiro S, Robinson A, Ducruet T, Huynh T (2010). Smoked cannabis for chronic neuropathic pain: a randomized controlled trial. CMAJ.

[6] Abrams DI, Jay CA, Shade SB, Vizoso H, Reda H, Press S (2007). Cannabis in painful HIV-associated sensory neuropathy: a randomized placebo-controlled trial. Neurology.

[7] Russo EB (2008). Cannabinoids in the management of difficult to treat pain. Ther Clin Risk Manag.

[8] Kowal MA, Hazekamp A, Grotenhermen F. Review on clinical studies with cannabis and cannabinoids 2010–2014. Cannabinoids. 2016;11:1–18.

[9] Kim JH, Santaella-Tenorio J, Mauro C, Wrobel J, Cerdà M, Keyes KM (2016). State medical marijuana laws and the prevalence of opioids detected among fatally injured drivers. Am J Public Health.

[10] Wen H, Hockenberry JM (2018). Association of medical and adult-use marijuana laws with opioid prescribing for medicaid enrollees. JAMA Intern Med.

[11] Morris RG, TenEyck M, Barnes JC, Kovandzic TV (2014). The effect of medical marijuana laws on crime: evidence from state panel data, 1990-2006. PLoS One.

[12] Anderson DM, Rees DI, Sabia JJ (2014). Medical marijuana laws and suicides by gender and age. Am J Public Health.

[13] Anderson DM, Hansen B, Rees DI. Medical marijuana laws, traffic fatalities, and alcohol consumption. J law Econ 2013;56(2):333–369. Available from: http://www.econstor.eu/handle/10419/58536%5Cnhttp://www.jstor.org/stable/10.1086/668812

[14] Santaella-Tenorio J, Mauro CM, Wall MM, Kim JH, Cerdá M, Keyes KM (2017). US traffic fatalities, 1985-2014, and their relationship to medical marijuana laws. Am J Public Health.

[15] Asbridge M, Hayden JA, Cartwright JL. Acute cannabis consumption and motor vehicle collision risk: systematic review of observational studies and meta-analysis. BMJ [Internet]. British Medical Journal Publishing Group. 2012;344:e536. Available from: http://www.ncbi.nlm.nih.gov/pubmed/22323502. [cited 18 Jan 2019]. 10.1136/bmj.e536 PMC3277079 22323502

[16] Fischer B, Russell C, Sabioni P, van den Brink W, Le Foll B, Hall W (2017). Lower-risk cannabis use guidelines: a comprehensive update of evidence and recommendations. Am J Public Health.

[17] Hashibe M, Morgenstern H, Cui Y, Tashkin DP, Zhang ZF, Cozen W (2006). Marijuana use and the risk of lung and upper aerodigestive tract cancers: results of a population-based case-control study. Cancer Epidemiol Biomark Prev.

[18] Morris MA, Jacobson SR, Kinney GL, Tashkin DP, Woodruff PG, Hoffman EA (2018). Marijuana use Associations with pulmonary symptoms and function in tobacco smokers enrolled in the subpopulations and intermediate outcome measures in COPD study (SPIROMICS). Chronic Obstr Pulm Dis.

[19] Aldington S, Harwood M, Cox B, Weatherall M, Beckert L, Hansell A (2008). Cannabis use and risk of lung cancer: a case-control study. Eur Respir J.

[20] Callaghan RC, Allebeck P, Sidorchuk A (2013). Marijuana use and risk of lung cancer: a 40-year cohort study. Cancer Causes Control.

[21] Y-HJ H, Zhang Z-F, Tashkin DP, Feng B, Straif K, Hashibe M (2015). An epidemiologic review of marijuana and cancer: an update. Cancer Epidemiol Biomarkers Prev.

[22] Tashkin DP (2013). Effects of marijuana smoking on the lung. Ann Am Thorac Soc.

[23] Conner SN, Bedell V, Lipsey K, Macones GA, Cahill AG, Tuuli MG (2016). Maternal marijuana use and adverse neonatal outcomes: a systematic review and meta-analysis. Obstet Gynecol.

[24] Westfall RE, Janssen PA, Lucas P, Capler R (2009). Reprint of: survey of medicinal cannabis use among childbearing women: patterns of its use in pregnancy and retroactive self-assessment of its efficacy against “morning sickness.”. Complement Ther Clin Pract.

[25] Ammerman S, Ryan S, Adelman WP, Committee on Substance Abuse, the Committee on Adolescence (2015). The impact of marijuana policies on youth: clinical, research, and legal update. Pediatrics.

[26] DiNardo J, Lemieux T (2001). Alcohol, marijuana, and American youth: the unintended consequences of government regulation. J Health Econ.

[27] Cohen M, Solowij N, Carr V (2008). Cannabis, cannabinoids and schizophrenia: integration of the evidence. Aust N Z J Psychiatry.

[28] Holdcroft A (2000). Adverse effects of cannabis and cannabinoids. British J Anaesthesia.

[29] Walsh Z, Gonzalez R, Crosby K, Thiessen M, Carroll C, Bonn-Miller MO. Medical cannabis and mental health: a guided systematic review. Clin Psychol Rev. 2016;51:15–29. 10.1016/j.cpr.2016.10.002 27816801

[30] Downey LA, King R, Papafotiou K, Swann P, Ogden E, Boorman M (2013). The effects of cannabis and alcohol on simulated driving: Influences of dose and experience. Accid Anal Prev.

[31] Perreault S. Impaired Driving in Canada, 2015 [Internet]. Ottawa, ON; 2016. Available from: https://www150.statcan.gc.ca/n1/en/pub/85-002-x/2016001/article/14679-eng.pdf?st=iIxKiIZt.

[32] Nutt DJ, King LA, Phillips LD, Montaner J, Wood E, Independent Scientific Committee on Drugs T (2010). Drug harms in the UK: a multicriteria decision analysis. Lancet.

[33] Rehm J, Baliunas D, Brochu S, Fischer B, Gnam W, PAtra J, Popova S, Sarnocinska-Hart A, Taylor B. The costs of substance abuse in Canada 2002. Ottawa. p. 2006. 10.15288/jsad.2007.68.886 17960307

[34] Stockwell T, Vallance K, Martin G, Macdonald S, Ivsins A, Chow C, et al. The price of getting high , stoned and drunk in BC: A comparison of minimum prices for alcohol and other psychoactive substances; 2010. p. 1–8. Available from: http://citeseerx.ist.psu.edu/viewdoc/download?doi=10.1.1.688.9654&rep=rep1&type=pdf

[35] Wood E, Werb D, Marshall BD, Montaner JS, Kerr T (2009). The war on drugs: a devastating public-policy disaster. Lancet.

[36] Wood E, McKinnon M, Strang R, Kendall PR (2012). Improving community health and safety in Canada through evidence-based policies on illegal drugs. Open Med.

[37] Walsh Z, Callaway R, Belle-Isle L, Capler R, Kay R, Lucas P (2013). Cannabis for therapeutic purposes: patient characteristics, access, and reasons for use. Int J Drug Policy.

[38] Lucas P, Walsh Z. Medical cannabis access, use, and substitution for prescription opioids and other substances: A survey of authorized medical cannabis patients. Int J Drug Policy. 2017;42:30–5. 10.1016/j.drugpo.2017.01.011 28189912

[39] Bottorff JL, Bissell LJL, Balneaves LG, Oliffe JL, Kang HBK, Capler NR, et al. Health effects of using cannabis for therapeutic purposes: a gender analysis of users’ perspectives. Subst Use Misuse. 2011;46(6):769–80. 10.3109/10826084.2010.537732 21138343

[40] Lucas P (2009). Moral regulation and the presumption of guilt in Health Canada’s medical cannabis policy and practice. Int J Drug Policy..

[41] Belle-Isle L, Walsh Z, Callaway R, Lucas P, Capler R, Kay R (2014). Barriers to access for Canadians who use cannabis for therapeutic purposes. Int J Drug Policy.

[42] Health Canada Cannabis Market Data (2018). Cannabis Market Data.

[43] Lucas P, Walsh Z, Crosby K, Callaway R, Belle-Isle L, Kay R (2016). Substituting cannabis for prescription drugs, alcohol and other substances among medical cannabis patients: the impact of contextual factors. Drug Alcohol Rev.

[44] StatsCan (2011). Canadian Census 2011.

[45] Reiman A, Welty M, Solomon P (2017). Cannabis as a substitute for opioid-based pain medication: patient self-report. Cannabis Cannabinoid Res.

[46] Lucas P (2012). Cannabis as an adjunct to or substitute for opiates in the treatment of chronic pain. J Psychoactive Drugs.

[47] Walsh Z, Gonzalez R, Crosby KS, Thiessen M, Carroll C, Bonn-Miller MO (2017). Medical cannabis and mental health: a guided systematic review. Clin Psychol Rev.

[48] DeRosse P, Kaplan A, Burdick KE, Lencz T, Malhotra AK (2010). Cannabis use disorders in schizophrenia: effects on cognition and symptoms. Schizophr Res.

[49] Katzman MA, Furtado M, Anand L (2016). Targeting the endocannabinoid system in psychiatric illness. J Clin Psychopharmacol.

[50] Sagar KA, Dahlgren MK, Racine MT, Dreman MW, Olson DP, Gruber SA (2016). Joint effects: a pilot investigation of the impact of bipolar disorder and marijuana use on cognitive function and mood. Hashimoto K, editor. PLoS One.

[51] Werb D, Watson TM, Maghsoudi N (2015). State of the evidence; cannabis use and regulation.

[52] Boyd S. Drug use, arrests, policing, and imprisonment in Canada and BC, 2015–2016. Vancouver; 2018. Available from: https://drugpolicy.ca/wp-content/uploads/2018/03/Vandu-Report-Mar-9-2018.pdf. [cited 2018 Sep 11]

[53] Opioid-Related Harms in Canada. Ottawa; 2017 Available from: www.cihi.ca. [cited 2018 Sep 11]

[54] Bradford AC, Bradford WD. Medical marijuana laws may be associated with a decline in the number of prescriptions for medicaid enrollees. Health Aff 2017;36(5):945–951. Available from: http://www.healthaffairs.org/doi/10.1377/hlthaff.2016.1135. [cited 2018 Jun 13] 10.1377/hlthaff.2016.1135 28424215

[55] Lucas P (2017). Rationale for cannabis-based interventions in the opioid overdose crisis. Harm Reduct J.

[56] Boehnke KF, Litinas E, Clauw DJ, Arnold LM, Clauw DJ, Dunegan LJ (2016). Medical cannabis use is associated with decreased opiate medication use in a retrospective cross-sectional survey of patients with chronic pain. J Pain.

[57] Reddon H, DeBeck K, Socias ME, Dong H, Wood E, Montaner J (2018). Cannabis use is associated with lower rates of initiation of injection drug use among street-involved youth: a longitudinal analysis. Drug Alcohol Rev.

[58] Scavone JL, Sterling RC, Weinstein SP, Van Bockstaele EJ (2013). Impact of cannabis use during stabilization on methadone maintenance treatment. Am J Addict.

[59] Raby WN, Carpenter KM, Rothenberg J, Brooks AC, Jiang H, Sullivan M (2009). Intermittent marijuana use is associated with improved retention in naltrexone treatment for opiate-dependence.

[60] Socías ME, Wood E, Lake S, Nolan S, Fairbairn N, Hayashi K (2018). High-intensity cannabis use is associated with retention in opioid agonist treatment: a longitudinal analysis. Addiction.

[61] Eysenbach G (2004). Improving the quality of web surveys: the checklist for reporting results of internet E-surveys (CHERRIES) | Eysenbach | Journal of Medical Internet Research. J Med Internet Res.

